# Analysis of Cell Signal Transduction Based on Kullback–Leibler Divergence: Channel Capacity and Conservation of Its Production Rate during Cascade

**DOI:** 10.3390/e20060438

**Published:** 2018-06-05

**Authors:** Tatsuaki Tsuruyama

**Affiliations:** 1 Clinical Research Center for Medical Equipment Development, Kyoto University Hospital, Shogoin-Kawahara-cho 54, Sakyo-ku, Kyoto 606-8057, Japan; tsuruyam@kuhp.kyoto-u.ac.jp; Tel.: +81-75-366-7694; Fax: +81-75-366-7660; 2 Department of Drug Discovery Medicine, Pathology Division, Graduate School of Medicine, Kyoto University, Yoshida-Konoe-cho, Sakyo-ku, Kyoto 606-8315, Japan

**Keywords:** average entropy production rate, fluctuation theorem, signal transduction

## Abstract

Kullback–Leibler divergence (KLD) is a type of extended mutual entropy, which is used as a measure of information gain when transferring from a prior distribution to a posterior distribution. In this study, KLD is applied to the thermodynamic analysis of cell signal transduction cascade and serves an alternative to mutual entropy. When KLD is minimized, the divergence is given by the ratio of the prior selection probability of the signaling molecule to the posterior selection probability. Moreover, the information gain during the entire channel is shown to be adequately described by average KLD production rate. Thus, this approach provides a framework for the quantitative analysis of signal transduction. Moreover, the proposed approach can identify an effective cascade for a signaling network.

## 1. Introduction

Kullback–Leibler divergence (KLD) is a type of generalized entropy or information quantity. It was introduced by Solomon Kullback and Richard A. Leibler, who discussed information source coding theory for information transmission efficiency [1]. At present, KLD finds diverse applications, including the imaging analytical field [2,3], hydrodynamics [4], clinical laboratory tests including electrocardiogram [5,6], network analysis [7,8] for biological applications [9], cellular biology [10], evaluating the bioequivalence of formulations of a drug [11], and experimental design for clinical study [12]. In this study, KLD is applied to analyze the cell information transmission, signal transduction, mediated by the cellular biochemical reaction. In particular, the proposed approach using Bayesian statistics [13,14], which is based on KLD, is expected to provide a novel theoretical framework [7].

Previous studies have discussed cell signal transduction through pathways from a viewpoint of similarity in the thermodynamic process that produces entropy [15]. Luo et al. [16] analyzed the heat production during carbohydrate metabolism and estimated the relationship between energy consumption and biological information from a biological metabolism perspective. Moreover, several research studies have applied variable concepts of entropy. In biologic genome informatics, a type of expanded Shannon entropy, such as the local Shannon-Jayne entropy, is utilized for analyzing the correlation between a set of gene expressions [17,18,19,20,21,22]. Teschendorff et al. [20] introduced a stochastic matrix, whose components are the normalized probabilities of the gene expressions in individual samples; the signal entropy rate was obtained using the matrix. In the network, the maximum entropy rate is determined by the adjacency matrix of the network [20]. For a multi-cell system, the network approach is useful for understanding biological signal transduction behaviors [23,24,25]. Recently, single cell entropy was introduced as the analytical basis of the variable phenotypic or genotypic state of a single cell, which is based on the assembly framework of statistical physics [26]. In another study, using a non-Markovian approach, the mutual entropy between the stimulus and the response in a biological system was considered in a sensory system, wherein the past trajectories are utilized to add useful information to the present state. In a simulation conducted by Becker et al., E. coli was used to reliably predict the concentration changes of environmental chemokines for chemotaxis [27].

Previously, the authors reported analyses of biological signal transduction based on information thermodynamics. In these studies, a theoretical framework was developed on Shannon entropy. However, the objective of the present study is to evaluate signal transduction efficiency of KLD in individual steps on the actual biochemical reaction kinetics [28,29]. KLD is introduced for analysis of signal transduction in reference to fluctuation theorem (FT) [30,31,32,33].

### Signal Cascade Model

The signal events can be modeled as a cascade of modification and/or demodification cycle reactions of proteins in a cell that are named signaling molecules. Equation (1) presents a signal cascade model [34,35]. Here, suffixes *m* and *j* represent the number of cascades and the step number, respectively. In this model, the signaling molecule at step 1 of cascade *m*, denoted by *X_m_*_1_, induces the modification of the *X_m_*_2_ into *X_m_*_2_* by binding the signal mediated molecule *A* such as adenosine triphosphate (ATP). Subsequently, *X_m_*_2_ activates *X_m_*_3_ in the same manner. In this way, the signaling molecule at the (*j* − 1)-th step of cascade *m*, denoted as *X_mj−_*_1_, induces the modification of *X_mj_* into X_mj_*. As the opposite orientation of signal, demodification of X_mj_* into *X_mj_* occurs, at the −(*j* − 1)-th step of cascade *m*, and the pre-stimulation steady state is subsequently recovered [34]:

(1) Xm1+Xm2+A↔Xm1+Xm2*  :1st&−1st step  Xmj−1*+Xmj+A↔Xmj−1*+Xmj* :j−1th &−(j−1)th step   Xm,n−1*+Xmn+A↔Xm,n−1*+Xmn*:n−1th &−(n−1)th step

In the above model, the subscript *m* represents the total number of the cascade.

We introduce *a priori* (prior) selection probability of signaling molecule for the analysis. Here, *q_mj_*, which represents the selection probability of inactive *X_mj_* used in the *j*-th step in cascade *m* (forward direction), takes the form of the *j*-th molecule. On the other hand, q_mj_*, which represents the selection probability of active X_mj_*, is used in the −*j*-th step for cascade *m* (backward direction), as follows:

(2) $$\begin{array}{l} q_{mj}=X_{mj}^{0}/X_{m}\\ q_{mj}*=X_{mj}^{0}*/X_{m} \end{array}$$

where,

(3) $$\sum_{j=1}^{n}\left(q_{mj}+q_{mj}*\right)=1$$

Here, *X_m_* indicates the total concentration of signaling molecules in cascade *m*:

(4) $$X_{m}=\sum_{j=1}^{n}\left(X_{mj}^{0}+X_{mj}^{0}*\right)$$

and the total concentration of active and inactive signaling molecules is given by:

(5) $$\sum_{j=1}^{n}X_{mj}^{0}=X_{m}$$

and

(6) $$\sum_{j=1}^{n}X_{mj}^{0}*=X_{m}*$$

The total duration of cascade *m*, *τ_m_^0^*, which indicates the sum of forward and backward cascades comprising a set of signaling molecules, is determined by:

(7) $$\tau_{m}^{0}=\sum_{j=1}^{n}\left(X_{m}^{0}\tau_{mj}^{0}-X_{m}^{0}*\tau_{-mj}^{0}\right)$$

In Equations (2), (5), (6) and (7), the total duration was determined using the probabilities *q_mj_* and q_mj_*:

(8) $$\tau_{m}^{0}=X_{m}\sum_{j=1}^{n}\left(q_{mj}\tau_{mj}^{0}-q_{mj}*\tau_{-mj}^{0}\right)$$

**Figure 1:**
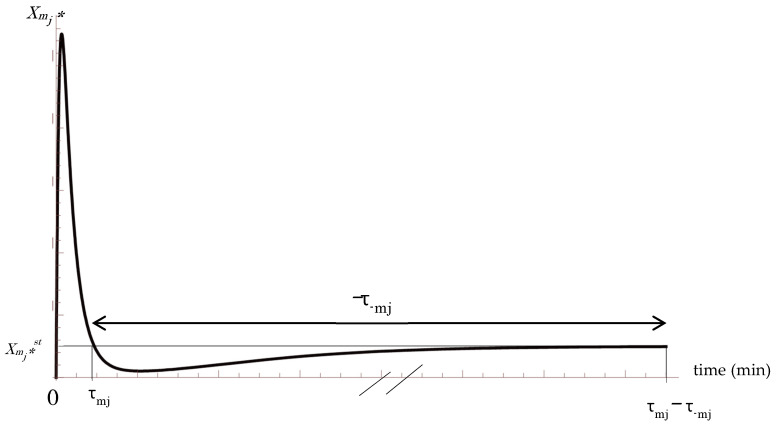
A common time course of the *j*-th step for both prior and posterior cascades, indicating concentration X_mj_*. The suffix 0 is omitted. The vertical axis denotes the concentration of signaling active molecule. *τ_mj_* and *τ_−mj_* represent the duration of the *j*-th step and the reverse *−j*-th step, respectively. The horizontal line X_mj_* = X_mj_*^st^ denotes the concentration of X_mj_* at the steady state [35]. The “//” symbol on the horizontal axis indicates *−τ_−mj_* or |*τ_−mj_| >> τ_mj_*.

The suffix 0 represents the prior state. Here, the duration, as forward *τ_mj_*^0^ and backward *τ_−mj_*^0^, are defined as shown in Figure 1. The Positive and negative values are assigned to *τ_mj_*^0^ and *τ_−mj_*^0^ corresponding to the direction of the step in the *m* cascade [34,35]. In the above equations, *τ_mj_*^0^ represents the duration corresponding to positive code length in which the active molecule X_mj_* increases in concentration. On the other hand, *τ_−mj_*^0^ represents the duration corresponding to negative code length in which the active molecule X_mj_* decreases in concentration. In this manner, the duration of individual step *j*-th can be represented as *τ_mj_*^0^ − *τ_−mj_*^0^.

## 2. Results

### 2.1. A Prior Probability Distribution of Signaling Molecules

Here, the author hypothesizes that the selection of signaling molecules is equal *a priori*. In our previous studies [34,35], Shannon’s entropy *H_m_* for *m*-th cascade was demonstrated. Using Equations (3), (5) and (6), entropy *H_m_*^0^ at *a priori* (prior) state can be represented as:

(9) $$H_{m}^{0}=-X_{m}\left(\sum_{j=1}^{n}q_{mj}\log q_{mj}+\sum_{j=1}^{n}q_{mj}*\log q_{mj}*\right)$$

To maximize *H_m_*^0^, using non-determined parameters *α_m_*^0^, and *β_m_*^0^, in reference to the constraints established by Equations (3) and (8), let us introduce a function *G*.

(10) $$\begin{array}{l} G\left(q_{m1},\;q_{m2},\;\cdots q_{mn};q_{m1}*,\;q_{m2}*,\;\cdots q_{mn}*;X_{m}\right)\\ =H_{m}^{0}\;-\;\alpha_{m}^{0}\sum_{j=1}^{n}\left(q_{mj}+q_{mj}*\right)\;-\;\beta_{m}^{0}\tau_{m}^{0}\\ =H_{m}^{0}\;-\;\alpha_{m}^{0}\sum_{j=1}^{n}\left(q_{mj}+q_{mj}*\right)\;-\;\beta_{m}^{0}X_{m}\sum_{j=1}^{n}(q_{mj}\tau_{mj}^{0}\;-\;q_{mj}*\tau_{-mj}^{0}) \end{array}$$

Then, we have

(11) $$\frac{\partial G}{\partial q_{mj}}=-X_{m}\left(\log q_{mj}-\beta_{m}^{0}\tau_{mj}^{0}\right)-\alpha_{m}^{0}-X_{m}$$

(12) $$\frac{\partial G}{\partial q_{mj}*}=-X_{m}\left(\log q_{mj}+\beta_{m}^{0}\tau_{-mj}^{0}\right)-\alpha_{m}^{0}-X_{m}$$

(13) $$\frac{\partial G}{\partial X_{m}}=-\left(\sum_{j=1}^{n}q_{mj}\log q_{mj}+q_{mj}*\log q_{mj}*\right)-\beta_{m}^{0}\sum_{j=1}^{n}\left(q_{mj}\tau_{mj}^{0}-q_{mj}*\tau_{-mj}^{0}\right)$$

To maximize *G*, the right sides of Equations (11)–(13) are equated to zero.

(14) $$-\log q_{mj}=\beta_{m}^{0}\tau_{mj}^{0}\;(\tau_{mj}^{0}\;>\;0)$$

(15) $$-\log q_{mj}*\;=-\beta_{m}^{0}\tau_{-mj}^{0}\;(\tau_{-mj}^{0}<0)$$

As indicated above, Equations (14) and (15) imply an important result; the coefficient *β_m_*^0^ is independent of the step number *j*. Therefore, Equations (14) and (15) will be utilized as a prior probability distribution later. Therefore, from Equations (9), (14) and (15) the author has:

(16) $$H_{m}^{0}=X_{m}\beta_{m}^{0}\tau_{m}^{0}$$

### 2.2. Average Entropy Production Rate in a Signal Cascade

Next, the kinetics were investigated using *q_m_* (*j*|*j* − 1), which is the transitional probability of the *j*-th given (*j* − 1)-th step, and *v_m_* (*j*|*j* − 1), which is the transitional rate of the *j*-th step in a forward signaling direction. Given *j*-th step, *q_m_* (*j* − 1|*j*) is the transitional probability of the (*j* − 1)-th step given step *j*-th step. Similarly, given *j*-th step, *v_m_* (*j* − 1|*j*) is the transitional rate of the (*j* − 1)-th step in a backward signaling direction in a given cascade. The cell system remains at detailed balance around the steady state, the *homeostasis*, as follows:

(17) $$q_{m}\left(j|j-1\right)v_{m}^{0}\left(j|j-1\right)=q_{m}\left(j-1|j\right)v_{m}^{0}\left(j-1|j\right)$$

Therefore, the author has:

(18) $$\log\frac{q_{m}\left(j-1|j\right)}{q_{m}\left(j|j-1\right)}=\log\frac{v_{m}^{0}\left(j|j-1\right)}{v_{m}^{0}\left(j-1|j\right)}$$

Using kinetic coefficients for (*j* − 1)-th step and the reverse −(*j* − 1)-th step, *k_m,j_*_−1_ and *k_m,_*_−(*j*−1)_ in (1), the right side of (18) is given

(19) $$\log\frac{q_{m}\left(j-1|j\right)}{q_{m}\left(j|j-1\right)}=\log\frac{v_{m}^{0}\left(j|j-1\right)}{v_{m}^{0}\left(j-1|j\right)}=\log\frac{k_{m,j-1}X_{m,j-1}^{0\;*}X_{mj}^{0}\;A}{k_{m,-(j-1)}X_{m,j-1}^{0\;*}X_{mj}^{0}*}=\log\frac{k_{m,j-1}X_{mj}^{0}A}{k_{m,-(j-1)}X_{mj}^{0}*}$$

When the change of *X_m,j_*_−1_* is negligible during signal transduction, relative to the fluctuation of X_mj_*, we have:

(20) $$\log\frac{q_{m}\left(j-1|j\right)}{q_{m}\left(j|j-1\right)}=\log\frac{k_{m,j-1}q_{mj}A}{k_{m,-(j-1)}q_{mj}*}$$

In above, we used Equation (2). Dividing the both sides of above by *τ_mj_* − *τ_−mj_* and taking the limit, the variables *q_mj_* and q_mj_* remain in the right side:

(21) $$\begin{array}{l} \underset{\tau_{mj}^{0}-\tau_{-mj}^{0}\to \infty }{\lim}\frac{1}{\tau_{mj}^{0}-\tau_{-mj}^{0}}\log\frac{q_{m}\left(j-1|j\right)}{q_{m}\left(j|j-1\right)}\\ =\underset{\tau_{mj}^{0}-\tau_{-mj}^{0}\to \infty }{\lim}\frac{1}{\tau_{mj}^{0}-\tau_{-mj}^{0}}\;\log\frac{q_{mj}}{q_{mj}*} \end{array}$$

Using Equations (14) and (15), the author has:

(22) $$\underset{\tau_{mj}^{0}-\tau_{-mj}^{0}\to \infty }{\lim}\frac{1}{\tau_{mj}^{0}-\tau_{-mj}^{0}}\;\log\frac{q_{m}\left(j-1|j\right)}{q_{m}\left(j|j-1\right)}=-\beta_{m}^{0}\left(\frac{\tau_{mj}^{0}+\tau_{-mj}^{0}}{\tau_{mj}^{0}-\tau_{-mj}^{0}}\;\right)\sim -\beta_{m}^{0}$$

(23) $$\underset{\left|-\tau_{mj}^{0}+\tau_{-mj}^{0}\right|->\infty }{\lim}\frac{1}{\tau_{mj}^{0}-\tau_{-mj}^{0}}\;\log\frac{q_{m}\left(j|j-1\right)}{q_{m}\left(j-1|j\right)}=\beta_{m}^{0}\left(\frac{\tau_{mj}^{0}+\tau_{-mj}^{0}}{\left|-\tau_{mj}^{0}+\tau_{-mj}^{0}\right|}\right)\sim \beta_{m}^{0}$$

In above Equations (22) and (23), |*τ*_−*mj*_^0^| is sufficiently longer than *τ*_*mj*_^0^, according to experimental studies (Figure 1) [36,37,38,39,40,41,42,43,44,45,46,]. Here, using an arbitrary time parameter *t*, the average entropy production rate (AEPR), 〈*ζ_mi_*〉 and 〈*ζ_−mi_*〉 are defined during signal transduction for *τ*_*mj*_^0^ − *τ*_−*mj*_^0^ and |*τ*_−*mj*_^0^ − *τ*_*mj*_^0^|. respectively for *m* cascade and reverse cascade −*m*.

(24) $$\langle \zeta_{mj}\rangle ≜\frac{1}{\tau_{mj}^{0}-\tau_{-mj}^{0}}\;\int_{0}^{\tau_{mj}^{0}-\tau_{-mj}^{0}}\zeta_{mj}\left(t_{mj}\right)dt_{mj}$$

(25) $$\langle \zeta_{-mj}\rangle ≜\frac{1}{\left|\tau_{mj}^{0}-\tau_{-mj}^{0}\right|}\int_{0}^{\left|\tau_{mj}^{0}-\tau_{-mj}^{0}\right|}\zeta_{-mj}\left(t_{-mj}\right)dt_{-mj}$$

The fluctuation theorem (FT) states that the right sides of Equations (22)–(25) are equal to AEPR

(26) $$\underset{\tau_{mj}^{0}-\tau_{-mj}^{0}\to \infty }{\lim}\frac{1}{\tau_{mj}^{0}-\tau_{-mj}^{0}}\log\frac{q_{m}\left(j|j-1\right)}{q_{m}\left(j-1|j\right)}=\langle \zeta_{mj}\rangle$$

(27) $$\underset{\left|-\tau_{mj}^{0}+\tau_{-mj}^{0}\right|->\infty }{\lim}\frac{1}{\left|\tau_{mj}-\tau_{-mj}\right|}\log\frac{q_{m}\left(j-1|j\right)}{q_{m}\left(j|j-1\right)}=\langle \zeta_{-mj}\rangle$$

and

(28) $$\beta_{m}^{0}=-\langle \zeta_{mj}\rangle =\langle \zeta_{-mj}\rangle$$

Here, *β_m_^0^* has the dimension of entropy production rate and AEPRs are independent of the step number. Subsequently, AEPRs are redefined using Equations (14), as follows:

(29) $$-\langle \zeta_{-mj}\rangle ≜\langle \zeta_{m}\rangle =-\frac{\log q_{mj}\;}{\tau_{mj}^{0}}$$

Notably, Equations (14), (15) and (28) indicate that AEPR is consistent during signal cascade. Here, the channel capacity is given by AEPR.

(30) $$-\log q_{mj}=\langle \zeta_{m}\rangle \tau_{mj}^{0}$$

(31) $$\log q_{mj}*=\langle \zeta_{m}\rangle \tau_{-mj}^{0}$$

In previous our studies, a simple formulation was proposed between selection probability *q_mj_* and duration *τ_mj_*, using an arbitrary parameter, ζ, which was independent of step numbers [34,35].

### 2.3. Multinomial Distribution with Population Distribution

KLD was used as a measure of information gain when obtaining *a posteriori* (posterior) distribution from the prior distribution in Equations (30) and (31) to a posterior distribution. Let probability *q_mj_* be the prior distribution. Therefore, the uncertainty reduces:

(32) $$\mathrm{Uncertainty}=-\log p_{mj}-\left(-\log q_{mj}\right)$$

We define information *D_m_*, as the KLD of signal events in cascade *m*,

(33) $$D_{m}\left(p_{m}\|q_{m}\right)=-X_{m}\sum_{j=1}^{n}\left(p_{mj}\log\frac{p_{mj}}{q_{mj}}+p_{mj}*\log\frac{p_{mj}*}{q_{mj}*}\right)$$

The above equation represents KLD, which indicates the average value of the information obtained from data *I* with respect to *p_mj_* and p_mj_*. Consequently, KLD is known as information gain. The maximum likelihood estimation is thought to be an estimation method that empirically minimizes KLD.

Posterior probabilities are defined:

(34) $$\sum_{j=1}^{n}\left[p_{mj}+p_{mj}*\right]=\;1\;$$

and

(35) $$\begin{array}{l} p_{mj}=X_{mj}/X_{m}\\ p_{mj}*=X_{mj}*/X_{m} \end{array}$$

In addition,

(36) $$\tau_{m}\;=X_{m}\sum_{j=1}^{n}\left(q_{mj}\tau_{mj}-q_{mj}*\tau_{-mj}\right)$$

Therefore, when considering the signal transduction occurs under a certain given condition, the probability is transformed from q_mj_* into p_mj_* with minimum KLD under the given condition.

To minimize *D_m_* (*p_mj_*||*q_mj_*) using non-determined parameters *α_m_*, and *β_m_* in reference to the constraints established by Equations (34) and (36), a function *L* was introduced to apply Lagrange’s method to undetermined multipliers.

(37) $$\begin{array}{l} L\left(p_{m1},\;p_{m2},\;\cdots p_{mn};p_{m1}*,\;p_{m2}*,\;\cdots p_{mn}*;X_{m}\right)\\ =D_{m}\left(p_{m}\|q_{m}\right)-\alpha_{m}\sum_{j=1}^{n}\left(p_{mj}+p_{mj}*\right)-\beta_{m}\tau_{m} \end{array}$$

Here, the differences between *α_m_* and *α_m_*^0^ and *β_m_*^0^ and *β_m_* are indicative of the signaling. Subsequently,

(38) $$\frac{\partial L}{\partial p_{mj}}=-X_{m}\left(\log\frac{p_{mj}}{q_{mj}}+\beta_{m}\tau_{mj}\right)-\alpha_{m}-X_{m}$$

(39) $$\frac{\partial L}{\partial p_{mj}*}=-X_{m}\left(\log\frac{p_{mj}*}{q_{mj}*}-\beta_{m}\tau_{-mj}\right)-\alpha_{m}-X_{m}$$

(40) $$\frac{\partial L}{\partial X_{m}}=-\left(\sum_{j=1}^{n}p_{mj}\log\frac{p_{mj}}{q_{mj}}+\sum_{j=1}^{n}p_{mj}*\log\frac{p_{mj}*}{q_{mj}*}\right)-\beta_{m}\left(\sum_{j=1}^{n}\tau_{mj}p_{mj}-\sum_{j=1}^{n}\tau_{-mj}p_{mj}*\right)$$

For the minimization of *L*, the right hand sides of Equations (38)–(40) are equated to zero, as follows:

(41) $$-\log\frac{q_{mj}}{p_{mj}}\;=\beta_{m}\tau_{mj}\;(\tau_{mj}>0)$$

(42) $$-\log\frac{q_{mj}*}{p_{mj}*}\;=-\beta_{m}\tau_{-mj}(\tau_{-mj}<0)$$

Therefore, from (41) and (42), the author has

(43) $$-\log p_{mj}=\beta_{m}\tau_{mj}+\beta_{m}^{0}\tau_{mj}$$

(44) $$-\log p_{mj}*=-\beta_{m}\tau_{-mj}-\beta_{m}^{0}\tau_{-mj}$$

(45) $$\alpha_{m}=-X_{m}$$

Accordingly, from Equations (7), (43) and (44), KLD is given by:

(46) $$\begin{array}{l} D_{m}\left(p_{m}\|q_{m}\right)=-X_{m}\sum_{j=1}^{n}\left(p_{mj}\log\frac{p_{mj}}{q_{mj}}+p_{mj}*\log\frac{p_{mj}*}{q_{mj}*}\right)\\ =X_{m}\beta_{m}\sum_{j=1}^{n}\left(p_{mj}\tau_{mj}-p_{mj}*\tau_{-mj}\right)\\ =\beta_{m}\tau_{m} \end{array}$$

And the author has:

(47) $$\beta_{m}=\frac{D_{m}\left(p_{m}\|q_{m}\right)}{\tau_{m}}=\delta_{m}\left(p_{m}\|q_{m}\right)$$

Accordingly, *β_m_* is equal to the average KLD production rate, δ(*p_m_*‖*q_m_*), during the signal transduction and is consistent during the entire signal cascade. Therefore,

(48) $$-\log\frac{p_{mj}}{q_{mj}}\;=\frac{D_{m}\left(p_{m}\|q_{m}\right)}{\tau_{m}}\tau_{mj}(\tau_{mj}>0)$$

(49) $$-\log\frac{p_{mj}*}{q_{mj}*}\;=-\frac{D_{m}\left(p_{m}\|q_{m}\right)}{\tau_{m}}\tau_{-mj}(\tau_{-mj}<0)$$

The author defined *p_m_*(*j*|*j* − 1), which is the transitional probability of the *j*-th given (*j* − 1)-th step, and *v_m_*(*j*|*j* − 1), which is the transitional rate of the *j*-th step in a forward signaling direction. In addition, given *j*-th step, *p_m_*(*j* − 1|*j*) is the transitional probability of the (*j* − 1)-th step given step *j*-th step. Similarly, given the *j*-th step, *v_m_*(*j* − 1|*j*) is the transitional rate of the (*j* − 1)-th step in a backward signaling direction in a given cascade.

(50) $$p_{m}\left(j|j-1\right)v_{m}\left(j|j-1\right)=p_{m}\left(j-1|j\right)v_{m}\left(j-1|j\right)$$

Likewise from (18)–(20), dividing the both sides of above by *τ_mj_* − *τ_−__mj_* and taking the limit, we have:

(51) $$\begin{array}{l} \underset{\tau_{mj}-\;\tau_{-mj}\to \infty }{\lim}\frac{1}{\tau_{mj}-\tau_{-mj}}\log\frac{p_{m}\left(j-1|j\right)}{p_{m}\left(j|j-1\right)}\\ =\underset{\tau_{mj}-\;\tau_{-mj}\to \infty }{\lim}\frac{1}{\tau_{mj}-\tau_{-mj}}\log\frac{p_{mj}}{p_{mj}*} \end{array}$$

Further, Equations (47), (50) and (51) give

(52) $$\begin{array}{l} \underset{\tau_{mj}-\tau_{-mj}\to \infty }{\lim}\frac{1}{\tau_{mj}-\tau_{-mj}}\log\frac{p_{m}\left(j-1|j\right)/q_{m}\left(j-1|j\right)}{p_{m}\left(j|j-1\right)/q_{m}\left(j|j-1\right)}\\ =\underset{\tau_{mj}-\tau_{-mj}\to \infty }{\lim}\frac{1}{\tau_{mj}-\tau_{-mj}}\frac{p_{mj}}{p_{mj}*}\frac{q_{mj}}{q_{mj}*}\\ \sim \delta_{m}\left(p_{m}\|q_{m}\right) \end{array}$$

In above, the author used *τ_mj_* << *τ_−mj_* (Figure 1). Above Equation corresponds to the extended FT considering KLD, and the extended channel capacity can be defined as:

(53) $$C_{m}=\underset{\tau \to \infty }{\lim}\frac{KD_{m}\left(p_{m}\|q_{m}\right)}{\tau_{m}}\;=\underset{\tau \to \infty }{\lim}K\delta_{m}\left(p_{m}\|q_{m}\right)$$

Here, *K* is an arbitrary constant. If entropy unit is used, *K* = *k_B_*, Boltzmann’s constant. On the other hand, in information science, *K* is equivalent to log_2_*e*.

## 3. Conclusions

Recently, theoretical analysis of the transduction capacity of biochemical signaling networks has greatly developed [47]. In this study, KLD, the average KLD production rate, and channel capacity based on the average KLD production rate were shown to be critical quantities that can be attributed to the entire signal transduction step. This KLD is a tool to estimate entropy production from stationary trajectories [48]. 

**Figure 2:**
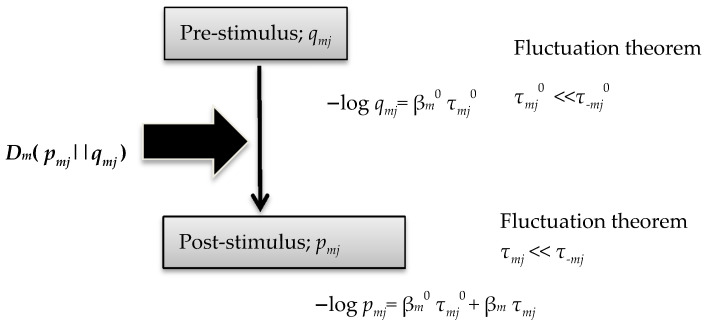
Theoretical framework of the current study.

In this work, the author deduced simple but important relational formulae, (47)–(53). For a prior distribution probability *q_mj_*, the author derived Equations (14) and (15) in association with FT and source-coding theory [28]. This method was introduced in our previous study of Tsallis entropy [29]. The theoretical framework in the current study is shown in Figure 2.

In this study, uniform distribution was not applied as a prior distribution *q_mj_* before stimulus. As reported previously in [34,35], the selection probability of signal molecules, *q_mj_*, can be described by simple formulae given by Equations (14) and (15), which illustrate the simple relationship between the logarithm of the probability and time elapsed between tentative modification and demodification. Subsequent to the stimulus, the selection probability of the signal molecules are transformed into a posterior distribution probability *p_mj_*. It is likely that the formulation of signal transformation using KLD is more intuitive and easier to understand than using mutual entropy. 

The Mitogen-activated Protein Kinase (MAPK) pathway is a multistep signal conversion step, in which the process of modification and demodification in the entire cascade can be understood as a repeated cycle reaction. It has been experimentally demonstrated that the demodification process is significantly longer than the modification process, as shown in *τ_mj_* << *τ_−mj_* (Figure 1), with the former requiring a few hours for completion, while the latter achieves completion in a few minutes. This asymmetry in the time course kinetics points to an important result pertaining the conservation of AEPR and average KLD production rate with reference to FT. In addition, it should be noted that in Equation (53), the channel capacity of the entire signal cascade is represented by KLD. Moreover, the author introduced average KLD production rate in Equation (47), and the production rate was found to be consistent during the whole cascade, which is an expanded form of AEPR conservation during the whole cascade, as reported previously by the author [35].

In the experimental studies [36,37,38,39,40,41,42,43,44,45,46], the ratio of modified active type of signaling molecules was determined by the immunoblot intensity or other corresponding data. Thus, it is possible to compute, in principle, the channel capacity of the entire signal cascade. This can be attributed to the fact that in most cases, extracellular substances, such as ligands, simultaneously promote the activation of multiple cascades. Therefore, the selection of specific ligands for the given cascade is essential and enables the measurement of the rigorous activity of the cascade. In future, such recombinant protein ligand will be required to quantify the signal cascade accurately.

Thus, KLD and average KLD production rate may be regarded as a critical attribution of cell signal cascade. The thermodynamic approach in this manuscript can provide a theoretical framework for the quantitative analysis of signal transduction.
